# Environmental Consortium Containing *Pseudomonas* and *Bacillus* Species Synergistically Degrades Polyethylene Terephthalate Plastic

**DOI:** 10.1128/mSphere.01151-20

**Published:** 2020-12-23

**Authors:** Cameron Roberts, Sabrina Edwards, Morgan Vague, Rosa León-Zayas, Henry Scheffer, Gayle Chan, Natasja A. Swartz, Jay L. Mellies

**Affiliations:** a Biology Department, Reed College, Portland, Oregon, USA; b Biology Department, Willamette University, Salem, Oregon, USA; c Chemistry Department, Reed College, Portland, Oregon, USA; University of Wisconsin-Madison

**Keywords:** PET plastic, pollution, bioaugmentation, *Pseudomonas*, *Bacillus*, consortia, biodegradation

## Abstract

Plastics, such as polyethylene terephthalate (PET) from water bottles, are polluting our oceans, cities, and soils. While a number of *Pseudomonas* species have been described that degrade aliphatic polyesters, such as polyethylene (PE) and polyurethane (PUR), few from this genus that degrade the semiaromatic polymer PET have been reported. In this study, plastic-degrading bacteria were isolated from petroleum-polluted soils and screened for lipase activity that has been associated with PET degradation. Strains and consortia of bacteria were grown in a liquid carbon-free basal medium (LCFBM) with PET as the sole carbon source. We monitored several key physical and chemical properties, including bacterial growth and modification of the plastic surface, using scanning electron microscopy (SEM) and attenuated total reflectance-Fourier transform infrared spectroscopy (ATR-FTIR) spectroscopy. We detected by-products of hydrolysis of PET using ^1^H-nuclear magnetic resonance (^1^H NMR) analysis, consistent with the ATR-FTIR data. The full consortium of five strains containing *Pseudomonas* and *Bacillus* species grew synergistically in the presence of PET and the cleavage product bis(2-hydroxyethyl) terephthalic acid (BHET) as sole sources of carbon. Secreted enzymes extracted from the full consortium were capable of fully converting BHET to the metabolically usable monomers terephthalic acid (TPA) and ethylene glycol. Draft genomes provided evidence for mixed enzymatic capabilities between the strains for metabolic degradation of TPA and ethylene glycol, the building blocks of PET polymers, indicating cooperation and ability to cross-feed in a limited nutrient environment with PET as the sole carbon source. The use of bacterial consortia for the biodegradation of PET may provide a partial solution to widespread planetary plastic accumulation.

**IMPORTANCE** While several research groups are utilizing purified enzymes to break down postconsumer PET to the monomers TPA and ethylene glycol to produce new PET products, here, we present a group of five soil bacteria in culture that are able to partially degrade this polymer. To date, mixed *Pseudomonas* spp. and *Bacillus* spp. biodegradation of PET has not been described, and this work highlights the possibility of using bacterial consortia to biodegrade or potentially to biorecycle PET plastic waste.

## Abstract (precis)

While several research groups are utilizing purified enzymes to break down postconsumer PET to the monomers TPA and ethylene glycol to produce new PET products, here, we present a group of five soil bacteria in culture that are able to partially degrade this polymer. To date, mixed *Pseudomonas* spp. and *Bacillus* spp. biodegradation of PET has not been described, and this work highlights the possibility of using bacterial consortia to biodegrade or potentially to biorecycle PET plastic waste.

## INTRODUCTION

Polyethylene terephthalate (PET) is among the most commonly produced polyesters worldwide and is used to produce water bottles and common household goods, such as carpet fibers, curtains, and fabrics. While most PET is recyclable, 60% of all postconsumer plastic produced globally accumulates either in landfills or the environment, posing a massive environmental problem (1). Chemically, PET plastic is composed of inert and hydrophobic repeating units of ethylene glycol and semiaromatic terephthalic acid (TPA) monomers linked by ester bonds. Due to chain length and its high proportion of semiaromatic components, PET is incredibly durable and stable and is resistant to environmental weathering which allows it to persist in the environment for many decades (2). Natural exposure to UV light and moisture makes plastic more susceptible to degradation (3), and as PET enters the environment, exposure to UV light and mechanical disruption may begin the initial process of degradation, resulting in micro- and nanoplastics that build up in food chains (4).

As plastics can be partially degraded by exposure to UV radiation, this pretreatment has been shown to increase carbonyl functionality via photooxidation. UV radiation greatly increased the ability of Brevibacillus borstelensis to degrade aliphatic polyethylene (PE) and for the ability of low-density polyethylene (LDPE) films to be degraded by a mixed culture of Lysinibacillus xylanilyticus and Aspergillus niger (5, 6). Spontaneous hydrolysis, photooxidation, and mechanical separation of plastic have been shown to enhance biodegradation by chemical and physical modification of the plastic surface (7). Research has even indicated that microbes may be able to degrade plastic without the use of UV or other forms of pretreatment (8–10). For example, the enzyme PETase, secreted by the bacterium Ideonella sakaiensis (2), can degrade the ester linkages in amorphous PET without pretreatment. Novel cutinases, found predominantly in fungal species such as Fusarium solani and Humicola insolens (11, 12), have also been shown to be active against PET plastic.

Other classes of polyesterases associated with lipase activity have been shown to act on PET (13). Lipase-positive bacteria have been associated with polymer degradation due to lipid and polymer chain structural similarities and bacterial capability of cleaving water-insoluble compounds (14). A well-studied lipase isolated from yeast species Candida antarctica (CALB) has been shown to singly depolymerize PET and catalyze the release of TPA as well as synergistically cleave TPA in combination with a cutinase from Humicola insolens (15). Once large, branched polymers are disassembled into monomers, oligomers, aldehydes, ketones, and other small molecules, they can be taken up by the cell and used as sources of energy, which is particularly useful for microbes residing in plastic-rich environments.

Contaminated environments containing PET are exposed to UV radiation and/or mechanical modification from the surrounding environment, and thus, microbial populations residing in those environments may have the ability to more readily degrade PET and to utilize resulting monomers. *Pseudomonas* and *Bacillus* species found in highly polluted environments may have developed the ability to degrade PET, as they are known to metabolize aromatic compounds and other polymeric materials (16–19). Further, both monomers of PET, TPA and ethylene glycol, are metabolized by some species of *Pseudomonas* and *Bacillus* (20–22) that are commonly found in soils. It is well established that petroleum-contaminated sites can contain significant sources of indigenous “hydrocarbon-degrading” bacteria (14, 23). As petroleum is rich in diverse chemical compounds and various aromatic hydrocarbons (24), we expected to find a mixture of these bacteria or others capable of degrading the semiaromatic polyester PET by sampling soils polluted with petroleum products, which contains both aliphatic and aromatic compounds.

## RESULTS

### Bacterial isolation and lipase screening.

**FIG 1:**
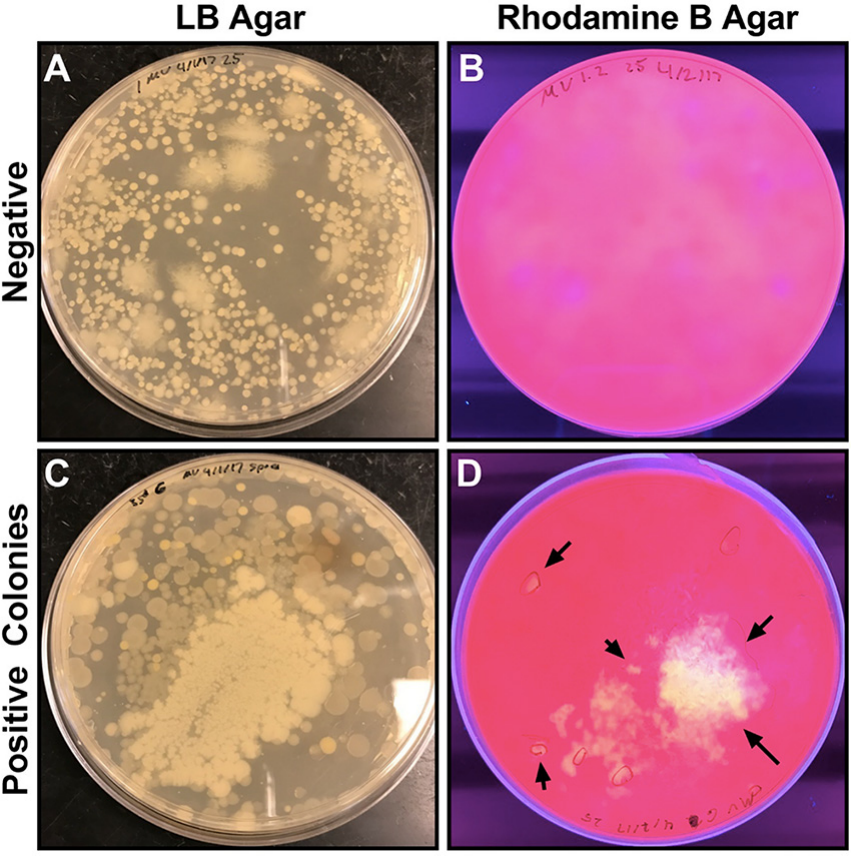
Rhodamine B agar screen for lipase activity. Master plates of mixed colonies were generated by soaking soil samples in water and collecting the supernatant to spread on LB plates. Individual plates with growth (A and C) were stamped onto rhodamine B plates (4.0% wt/vol) (B and D) to screen for lipase activity. The presence of orange or yellow halos under 365-nm UV exposure indicates lipase positive colonies (indicated with arrows). After, individual colonies in lipase-positive areas were spotted onto new rhodamine plates to isolate the lipase producers, and positive spots were restreaked onto LB agar for purification.

To screen soil samples for lipase activity, bacteria were plated on rhodamine B agar containing olive oil as a substrate. The presence of glowing halos, upon exposure to UV light, around colonies indicated lipase activity (Fig. 1D). Colonies that appeared lipase positive were isolated and retested to confirm lipase activity and to isolate pure cultures. In total, 192 colonies were screened after appearing lipase positive or growing near-lipase-positive bacteria during this screening.

**FIG 2:**
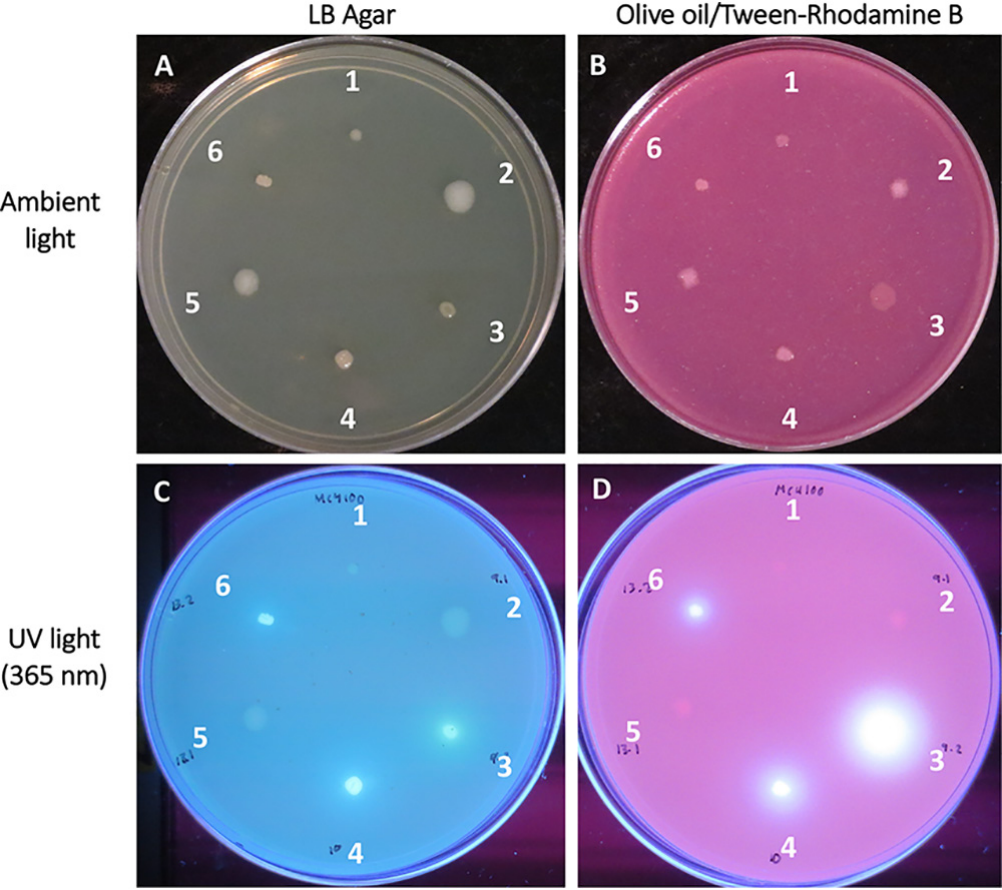
Lipase production by strains plated on rhodamine B agar containing olive oil demonstrating lipolytic activity. Lipase negative *E coli* strain MC4100 (1) was a negative control. Strains 9.1 (2), 9.2 (3), 10 (4), 13.1 (5), and 13.2 (6) were inoculated on LB and olive oil-rhodamine B plates grown for 48 h at 26°C without UV exposure (A and B) and with UV light at 365 nm (C and D). (C) LB plates show natural fluorescence. (D) The rhodamine B plate shows lipolytic activity indicated by white halos for strains 9.2, 10, and 13.2.

Gram staining indicated the cultures were mixed, with various morphologies, most likely as consortia when grown on LB agar. From consortium 9, two strains were purified, a Gram-negative rod (strain 9.2) and a Gram-positive rod (strain 9.1). From consortium 13, two strains were also purified, a Gram-negative rod (strain 13.2) and a Gram-positive rod (strain 13.1). Consortium 10 eventually had a single morphology by Gram stain, indicating purification to a single strain (strain 10). Pure cultures were confirmed for lipase activity using rhodamine B agar (Fig. 2). Strains 9.2, 10, and 13.2, all Gram-negative rods, tested positive for lipase activity, suggesting that they might be capable of plastic degradation (Fig. 2D). Strains 9.1 and 13.1 were lipase negative, as was the negative control, E. coli strain MC4100. Fluorescent halos, indicating lipase activity, on rhodamine B agar can be easily distinguished from colony growth and any natural fluorescence produced by pseudomonads (Fig. 2A and C). Because the halo observed for strain 9.2 was consistently larger than that of the other strains under UV light at 365 nm (Fig. 2D), the data suggested greater lipase activity for this strain than the other two lipase producers, strains 10 and 13.2.

Next, we determine whether the consortia could produce more lipase activity and potentially degrade plastic more rapidly than individual strains. To test this idea, we cultured consortium 9 and its individual strains taken from bacteria cultured on postconsumer PET plastic (see Materials and Methods) and then inoculated it onto rhodamine B plates to quantify lipase activities. Measuring the ratio of fluorescent halo to growth (25), consortium 9 possessed greater lipase activity than either strains 9.2 or 10 alone (*P* < 0.001; Table 1). These data indicated that strain 9.2 cultivated with strain 9.1 produced and/or secreted greater quantities of lipase than strains 9.2 or 10 alone.

**TABLE 1:** Consortium 9 synergistic lipase activity^*a*^

Strain(s)	Least square mean halo/growth (*n* = 5)	Student’s *t* test pairwise comparison to 10 (probability > |*t*|)	Student’s *t* test pairwise comparison to 9.2 (probability > |*t*|)
9.1	0	<0.001^*b*^	<0.001^*b*^
9.2	2.12 ± 0.04	0.0045^*b*^	
9.1, 9.2	2.98 ± 0.04	<0.001^*b*^	<0.001^*b*^
10	2.30 ± 0.04		0.0045^*b*^

a Strain 9.1 B. thuringiensis strain C15 and strain 9.2 *Pseudomonas* sp. B10 (consortium 9), strains 9.1, 9.2, or strain 10 *Pseudomonas* sp. SWI36 alone were swabbed from cultures with postconsumer PET as the sole carbon source and then inoculated onto rhodamine B plates with olive oil as a substrate (*n* = 5). The growth of each strain and the corresponding halo diameters were measured after 48 h. All diameters were quantified in ImageJ with a column average plot across each halo, and the ratio of halo to growth for each strain was compared to the two pseudomonads alone. By standard plate count, approximately equal numbers (∼200 CFU/ml) of strains 9.2 and 9.1, comprising consortium 9, were released from the PET plastic, upon gentle vortexing, after 8 weeks of incubation at room temperature. Strain 9.1 alone was unable to grow using PET as a sole source of carbon.

b *P* values < 0.05 were considered significant.

### General hydrolytic and polyesterase activities.

Due to the hydrolytic ester cleavage of PET releasing bis(2-hydroxyethyl) terephthalic acid (BHET) and mono(2-hydroxyethyl) terephthalic acid (MHET) (2), the major biodegradation by-products, we tested for general hydrolytic and polyesterase activity in all strains. The simple triglyceride tributyrin was used to screen for basic esterase activity. Positive activity was indicated by a clearing of substrate around colonies under normal lighting conditions. After growth at 21°C for 5 days, all strains exhibited the ability to cleave this small triglyceride (Table 2). As controls, Pseudomonas fluorescens (ATCC 49838) and Bacillus subtilis (ATCC 6051) were also positive for tributyrin clearing, whereas the negative control, Escherichia coli, did not clear the substrate (data not shown). Additionally, Tween 20 and Tween 80 were used as the substrates on calcium chloride agar to examine hydrolytic activities that fall between esterases and true lipases. Lipases act on water-insoluble substrates, such as triglycerides composed of long-chain fatty acids. Esterases preferentially hydrolyze simpler esters, typically shorter fatty acids, often containing fewer than 6 carbons. When incubated for 4 days at 26°C, of the five strains, only 9.1, 9.2, and 13.1 were positive for esterase activity (Table 2).

**TABLE 2:**
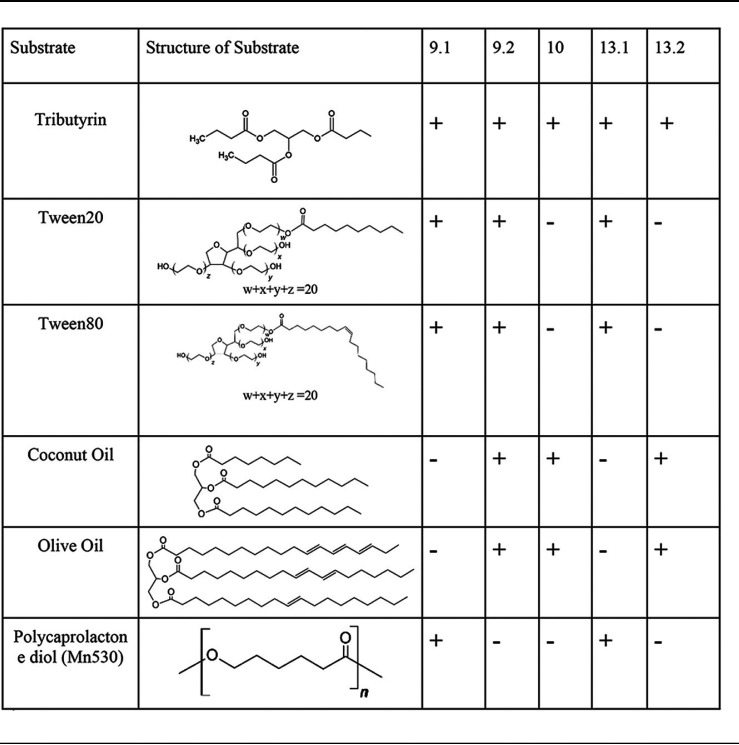
Substrates used for screening for various hydrolytic abilities of consortia strains^*a*^

a Short-, medium-, and long-chained substrates were used to assess specificity and promiscuity of esterase and lipase activities among strains. Simple triglyceride tributyrin was used to screen for basic ester cleavage. Tween 20 and Tween 80 were used to screen hydrolytic activities classified between esterases and true lipases. Coconut oil is a medium-chain fatty acid and was used to detect distinctly different lipase and esterase activities than those screened with Tween. Olive oil, a long-chain fatty acid, indicated true lipase activity. Polycaprolactone, a biodegradable polyester, was used to detect polymer hydrolysis/polyurethane esterase activities.

In contrast to the olive oil screen which was selective for lipases that cleave long-chain fatty acids, coconut oil/rhodamine B agar plates were utilized to observe medium-chain fatty acid lipase activity. Strains 9.2, 10, and 13.2 had bright halos when exposed to UV light after incubation at 26°C for 2 days, indicating a positive medium-chain lipase/esterase activity, which was consistent with the initial olive oil screening assay (Table 2; Fig. 2). Interestingly, the halos surrounding colonies of strain 13.2 were much larger than any other strain for the coconut oil assay (data not shown), paralleling the halo formation observed from 9.2 grown on olive oil (Fig. 2). Strain 9.2 was the only strain to possess both short-chain esterase activity and longer-chain lipase activity (Table 2).

Finally, the aliphatic polyester polycaprolactone diol (*M*_n_, 530) (PCD) was used as a substrate to assess polyesterase activity. Prolonged growth on PCD as a substrate at 21°C for 5 days showed distinct grainy accumulation of hydrolytic by-products around colonies of *Bacillus* strains 9.1 and 13.1, indicating only 9.1 and 13.1 were positive for aliphatic polyesterase activity (Table 2). However, despite the *Pseudomonas* strains 9.2, 10, and 13.2 having lipase activities shown in both the olive oil and coconut oil screens, they were unable to cleave PCD. In sum, all five strains had varied substrate clearance rates and hydrolytic abilities. Since consortium 9 exhibited greater lipase activity (Table 1), despite strain 9.1 being lipase negative, and all strains had mixed hydrolytic activities, there is evidence of an increased range of substrate specificity and degradative capabilities when strains were cultured together.

### Identification of consortia strains.

Draft genome sequences were obtained for the five strains at the Oregon State University (OSU) Center for Genome Research and Biocomputing, and the DNA sequences and genome assemblies were announced (26). The closest relatives of 16S rRNA genes were determined to be 9.1, Bacillus thuringiensis strain C15 (100% coverage, 100% identity); 9.2, *Pseudomonas* sp. strain B10 (100% coverage, 99% identity); 10, *Pseudomonas* sp. strain SWI36 (100% coverage, 100% identity); 13.1, Bacillus albus strain PFYN01 (100% coverage, 100% identity); and 13.2, *Pseudomonas* sp. strain SWI36 (100% coverage, 100% identity). The five axenic bacterial strains were deposited with the USDA NRRL Culture Collection.

### The full consortium grows synergistically on UV-treated PET and nontreated BHET.

**FIG 3:**
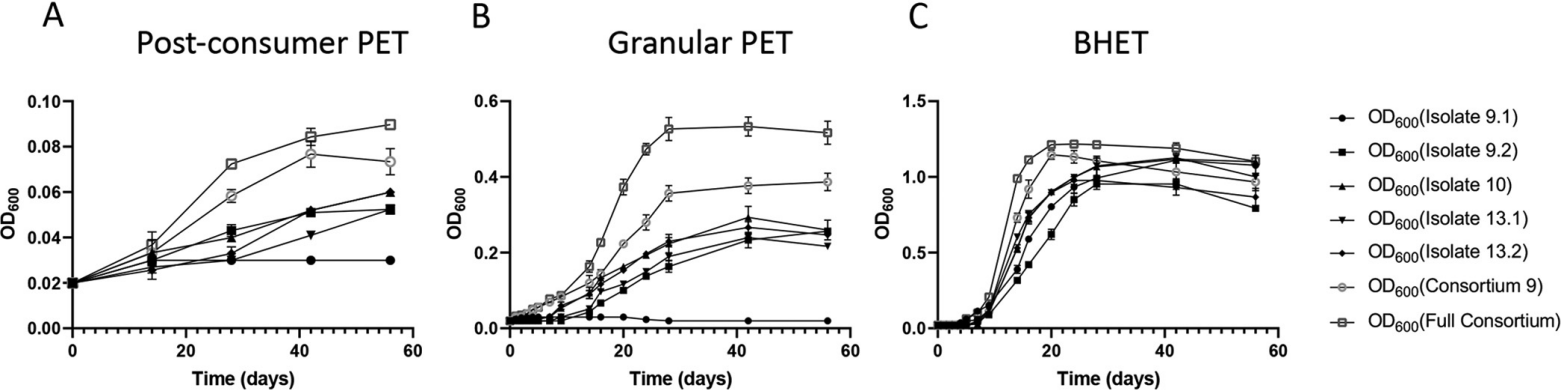
The full consortium grows faster, with higher yields on PET and BHET, than individual consortia or strains. Strains were grown on UV-treated postconsumer 2.0% wt/vol PET (A), 2.0% wt/vol UV-treated granular PET pellets (B), or 0.25% wt/vol BHET (C) as sole carbon sources. All strains were grown overnight in LCFBM supplemented with yeast extract and normalized by OD_600_ to ensure equal amounts of bacteria were added to all samples. Cultures containing PET were incubated statically, while those with BHET were shaken at 225 rpm at 30°C. Growth was measured every 24 h by an OD_600_ with a noninoculated sample as a blank. All experiments were performed in triplicate. Error bars indicate standard error.

The full consortium containing all bacterial strains had a shorter doubling time and greater carrying capacity on both UV-treated PET and BHET than individual strains (Fig. 3). All individual strains, consortia 9, and full consortium were grown with 2.0% (wt/vol) postconsumer PET, 2.0% (wt/vol) granular PET, or 0.25% (wt/vol) BHET in liquid carbon-free basal medium (LCFBM). All strains except 9.1 individually grew on postconsumer and granular PET. Control bacteria P. fluorescens (ATCC 49838) and B. subtilis (ATCC 6051) did not grow on this carbon source (data not shown). BHET, a by-product of partial hydrolysis of PET, was utilized by all five strains (Fig. 3).

**FIG 4:**
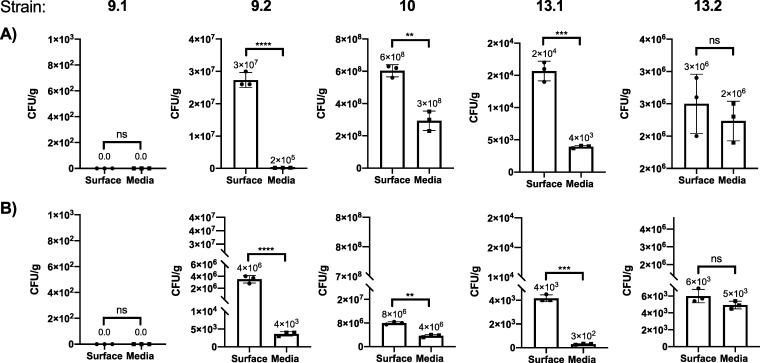
Differential growth of isolates on the surface of PET in LCFBM. All isolates were grown in LCFBM supplemented with granular (A) or postconsumer (B) PET for 6 weeks. By standard plate count, the total CFU/g of bacteria in the media were compared by Student's *t* test to CFU on the surface of substrate (*P* values < 0.05 were considered significant: *, *P* < 0.05; **, *P* < 0.01; ***, *P* < 0.001; and ****, *P* < 0.0001). Data are representative of three biological replicates, and means are reported above each bar with standard deviation.

We hypothesized that the five strains would differentially attach to the surface of PET because it is known that biofilms are necessary for efficient plastic degradation (10). We therefore determined the number of CFU per gram of plastic as a measure of attachment efficiency of each strain to PET. The total CFU per gram of plastic on the surface was greater than that found in the media for strains 9.2, 10, and 13.1 for both granular and postconsumer PET (Fig. 4). The CFU per gram on the surface of the plastic was 3 orders of magnitude greater than in the media for isolate 9.2 and 10-fold greater for isolate 13.1. Therefore, particularly strains 9.2 and 13.1 primarily grew on the surface of both granular and postconsumer PET.

### Surface degradation of PET.

**FIG 5:**
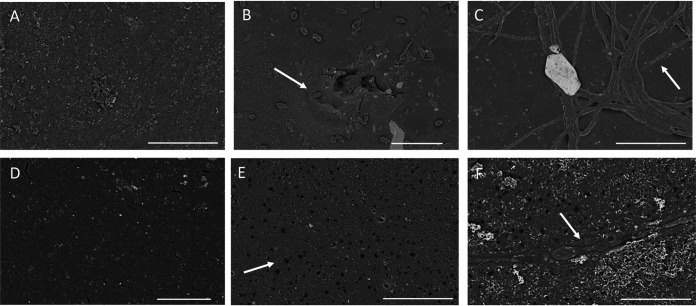
SEM images show surface modification of amorphous PET plastic. LCFBM containing amorphous PET and the full consortium were grown at 30°C for 40 days. Samples were treated with a mild detergent, saponin (A to C), to remove biofilms or with proteinase K (D to F) to remove remaining, tightly adherent bacteria prior to SEM. (A and D) Samples were uninoculated. Note, *Pseudomonas* (B) and *Bacillus* (C) indicated by arrows, and the cavities (E) and groove underlying lysed *Bacillus* bacteria (F) also indicated by arrows. Bars, 20 μm (A to C) and 5 μM (D to F).

To determine whether degradation could be observed on the PET surface, we first removed the bacteria by treating with the 0.1% saponin and then observed the surface of amorphous PET using scanning electron microscopy (SEM). As seen in Fig. 5, both *Pseudomonas* (Fig. 5B) and *Bacillus* (Fig. 5C) bacteria can still be observed tightly adherent to the surface of the plastic, even after treatment with the mild detergent, compared to the saponin-treated control that was not incubated with bacteria (Fig. 5A). Therefore, proteinase K (100 μg/ml) was used to remove all of the adherent bacteria in an attempt to observe physical alteration of the material. In Fig. 5E, we observed holes compared to the control, also treated with proteinase K but not incubated with bacteria (Fig. 5D). In Fig. 5F, we observed a bacillus along a trough that we interpreted as surface modification compared to the uninoculated control. Here, holes are also observed (Fig. 5F). Because both *Pseudomonas* and the *Bacillus* spp. are able to grow individually on PET as a sole source of carbon (Fig. 3), we concluded that surface modification of the amorphous PET was caused by both genera within the consortium.

### Secreted consortia enzymes converted BHET to TPA.

**FIG 6:**
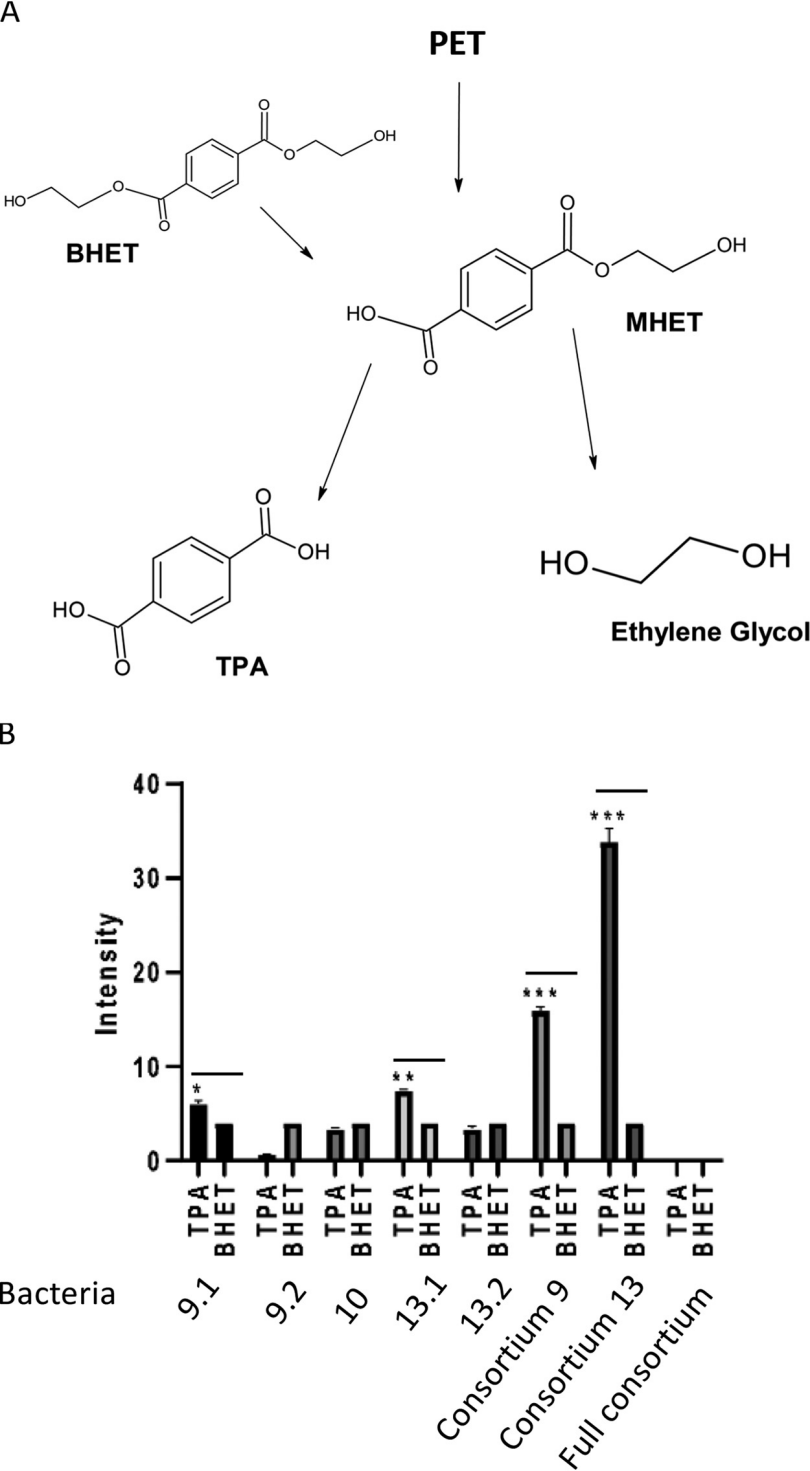
Secreted enzymes from the *Bacillus* species efficiently convert BHET to TPA. (A) PET degradation pathway. (B) Enzymes secreted by strains 9.1, 9.2, 10, 13.1, 13.2, and consortium 9, consortium 13, and the full consortium (FC) were added to 0.25% wt/vol BHET. Significant differences between TPA and BHET signals are represented as <0.05 (*), <0.01 (**), and <0.001 (***). The quantity of TPA from BHET cleavage was normalized by the presence of the BHET signal. For the full consortium, BHET was below detectable limits, suggesting there was near-complete conversion to TPA, unlike that observed for the other consortia and strains.

Cleavage of BHET to TPA and ethylene glycol (Fig. 6A) releases the monomers that can be taken up and metabolized by some soil bacteria or can be used as building blocks for polymerizing new PET plastic products. Thus, the enzymatic hydrolysis of the monomeric form of PET, BHET, was compared for natively secreted proteins from individual isolates and consortia. Consortia 9 and 13 significantly converted BHET to TPA (Fig. 6B). *Pseudomonas* individual strains 9.2, 10, and 13.2 had nonsignificant BHET to TPA conversion, whereas both *Bacillus* 9.1 and 13.1 strains had significant conversion levels. BHET quantities for the full consortium were below detectable limits, suggesting full conversion to TPA and ethylene glycol (Fig. 6B; Fig. S1 in the supplemental material). The secreted enzymes collected from cultures of the full consortium converted BHET to TPA with greater efficiency than enzymes collected from the cultures of individual isolates (Fig. 6B), and these results, combined with the observed growth (Fig. 3), indicated that the consortium also cooperatively degrades BHET.

### ATR-FTIR to detect surface modification.

**FIG 7:**
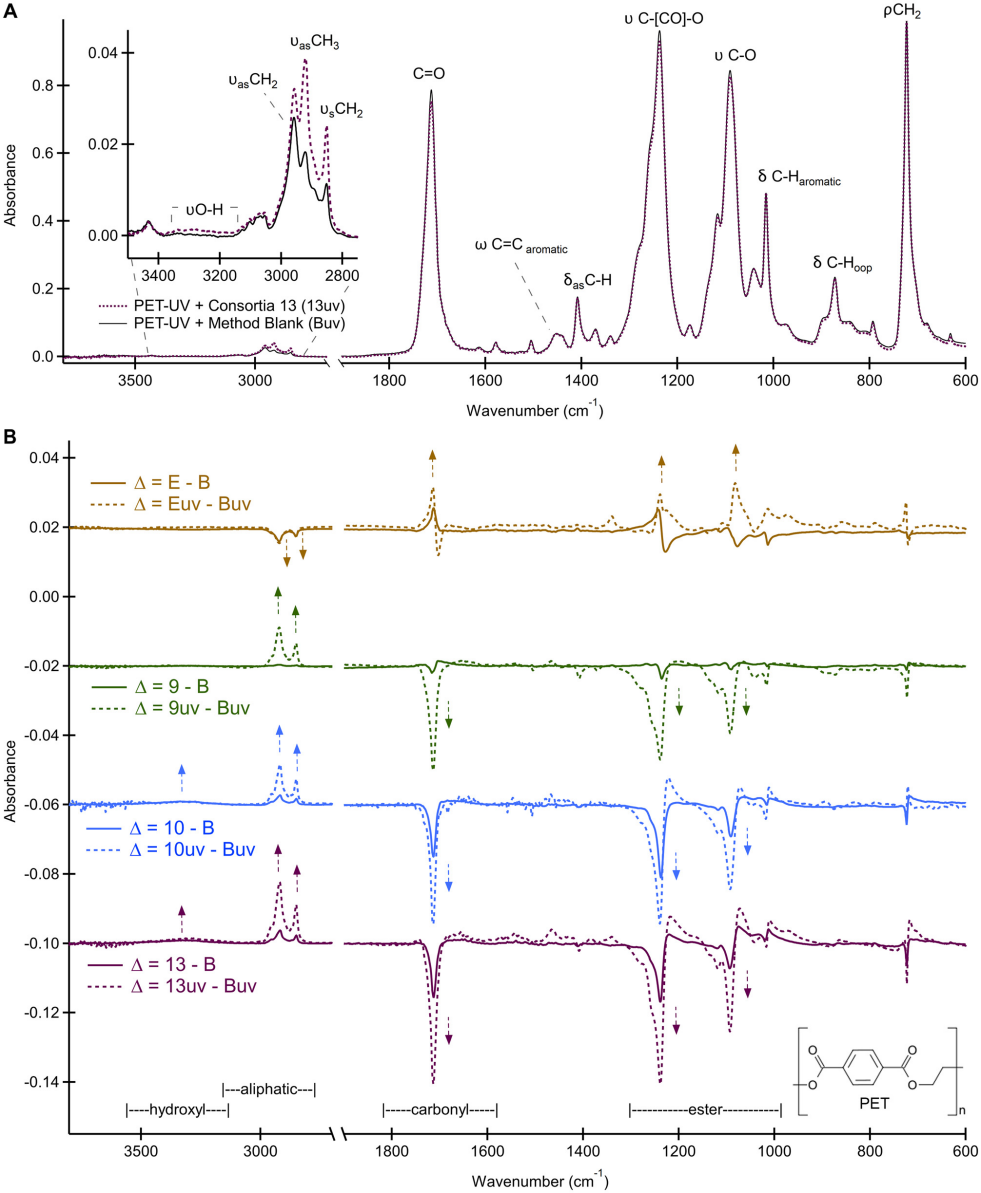
Infrared spectra of postconsumer PET plastics incubated with lipase positive consortia 9 and 13, strain 10, and E. coli. (A) Averaged ATR-FTIR spectrum acquired of the method blank (Buv) compared with consortium 13 (13uv) to illustrate representative peaks. (B) Comparison of PET difference spectra with UV (dashed line) and without UV (solid line) pretreatment prior to inoculation. Difference spectra (D) are vertically offset for clarity and were produced by spectral subtraction of the method blank (Buv) from the inoculated PET (9uv, 10uv, 13uv, or Euv). Direction of arrows indicates the growth or loss of a peak after incubation, signifying a relative increase or decrease in abundance of that bond, respectively. All spectra were averaged (*n* = 9) and normalized by peak area to the asymmetric bending mode (δ) at 1,408 cm^−1^ prior to spectral math.

Given that attachment and subsequent degradation are most likely to occur at the surface as opposed to the bulk material, the surface-sensitive technique attenuated total reflectance-Fourier transform infrared spectroscopy (ATR-FTIR) spectroscopy was used to monitor chemical changes of degraded plastics (27, 28). The averaged spectra collected for UV-irradiated postconsumer PET incubated with bacteria (13uv) and without (Buv) are shown in Fig. 7A, where major vibrational modes and respective chemical bonds of the polymer are identified. Difference spectra for PET (Fig. 7B) were calculated by subtraction of the averaged method blank spectrum (Buv) from each averaged treatment method spectrum (9uv, 10uv, 13uv, and Euv), and therefore, arrows indicate growth (↑) or loss (↓) of chemical bonds in the polymer.

As PET degrades, additional ester (C=O, C-O), carboxyl (C=O, C-O, O-H), or alcohol (C-O, OH) bonds are created in the polymer where the appearance or alteration of these bonds will change peak intensities at 2,958 cm^−1^, 1,713 cm^−1^, 1,089 cm^−1^, 888 cm^−1^, and 730 to 710 cm^−1^. Changes in polymeric bonds indicating degradation can be quantified by using an index calculated from the ratio of characteristic peak intensities to the normal C-H bending mode (at 1,409 cm^−1^). Using peak intensities, the relative amounts of aliphatic, carbonyl, and ester functionality of the treated plastic are listed in Table 3 for comparison of samples with and without inoculation or pretreatment by UV irradiation. Molecular changes calculated for samples without UV pretreatment were not statistically significant. Only the UV-pretreated PET samples showed substantial, reproducible spectral changes when analyzed by ATR-FTIR (Table 3; Fig. 7B).

**TABLE 3:** Molecular index and confidence intervals calculated by relative intensities of vibrational bands from ATR-FTIR analysis of commercial PET with and without UV pretreatment incubated in carbon-free medium for 6 weeks^*a*^

Sample type	Aliphatic index (*I*_2,958_/*I*_1,408_) with pretreatment:		Carbonyl index (*I*_1,713_/*I*_1,408_) with pretreatment:		Ester index (*I*_1,089_/*I*_1,408_) with pretreatment:	
	None	UV	None	UV	None	UV
Not treated	0.17 ± 0.01	0.17 ± 0.01	4.39 ± 0.05	4.34 ± 0.05	4.76 ± 0.04	4.64 ± 0.04
Blank^*b*^	0.17 ± 0.01	0.17 ± 0.01	4.45 ± 0.08	4.4 ± 0.1	4.74 ± 0.06	4.70 ± 0.09
E. coli^*c*^	0.17 ± 0.01	0.19 ± 0.01	4.50 ± 0.08	4.47 ± 0.09	4.75 ± 0.06	4.73 ± 0.06
Consortium 9	0.18 ± 0.04	0.25 ± 0.01	4.4 ± 0.1	4.1 ± 0.1	4.7 ± 0.1	4.50 ± 0.03
Strain 10	0.17 ± 0.01	0.30 ± 0.01	4.3 ± 0.1	4.05 ± 0.07	4.65 ± 0.07	4.37 ± 0.03
Consortium 13	0.18 ± 0.01	0.36 ± 0.01	4.3 ± 0.1	3.9 ± 0.4	4.68 ± 0.07	4.2 ± 0.3

a *n* = 9; *a* = 0.05.

b For the method blank, PET strips were incubated in carbon-free base medium without bacterial inoculate.

c For the experimental control, PET strips were incubated in carbon-free base medium and inoculated with E. coli strain MC4100.

Decreases in the carbonyl and ester indexes are expected during PET degradation, as C=O and C-O bonds are broken during ester cleavage. If undergoing chain scission of the polymer network, a simultaneous increase in the aliphatic index is expected as the abundance of saturated terminal ethylene glycol-like groups increase and midchain methylene bonds decrease. These changes indicating degradation of the plastic surface were observed for postconsumer PET cultured with consortia 9 and 13 and strain 10 (Fig. 7B; Table 3). Typical markers of plastic degradation by the bacteria were observed where the carbonyl index decreased systematically (9uv = 4.1 ± 0.1, 10uv = 4.05 ± 0.07, and 13uv = 3.9 ± 0.4 compared to Buv = 4.4 ± 0.1), as did the ester index (9uv = 4.50 ± 0.03, 10uv = 4.37 ± 0.03, and 13uv = 4.2 ± 0.3 compared to Buv = 4.70 ± 0.09) (Table 3).

All bacteria-inoculated PET samples presented a substantial increase in their aliphatic index (3,200 to 2,800 cm^−1^) (9uv = 0.25 ± 0.01, 10uv = 0.30 ± 0.01, and 13uv = 0.36 ± 0.01) compared to control samples (Buv = 0.17 ± 0.01) (Fig. 7B; Table 3). Loss of ester functionality was confirmed by the negative peaks in all difference spectra of PET (Fig. 7B), whereas the E. coli samples used as negative controls showed the inverse, a systematic increase in the same region between 1,300 and 1,000 cm^−1^. Even though small changes were observed in the averaged difference spectrum for Euv samples, no significant differences were found between carbonyl and ester indexes of blank and E. coli-incubated postconsumer PET samples (Tukey honestly significant difference [HSD], Euv to Buv; *P*_carbonyl_ = 0.946 and *P*_ester_ = 0.997). The aliphatic index of E. coli samples was observed to be significantly smaller than blank samples (Tukey HSD, Euv to Buv, *P*_aliphatic_ = 0.0356).

Postconsumer PET samples 9uv, 10uv, and 13uv were found to have substantially larger aliphatic indexes accompanied by a simultaneous decrease in carbonyl and ester indexes that suggest hydrolysis to smaller molecular fragments was occurring. These data indicate that spontaneous hydrolysis of PET in the carbon-free medium was not a favored mechanism during incubation and that any observed changes were instead due to inoculation with bacteria. Further, the decrease in the carbonyl and ester indices typically used as markers for plastic degradation were greatest in the UV-pretreated PET samples inoculated with bacteria, suggesting that UV pretreatment is necessary for the biodegradation of the postconsumer PET plastic.

### NMR spectra postinoculation resulted in detectable PET degradation products.

Next, we performed ^1^H-nuclear magnetic resonance (^1^H NMR) analysis to detect bacteria-driven changes of the surface and end groups of granular PET. After treatment with bacteria, PET samples were leached in dimethyl sulfoxide (DMSO)-d6, releasing small degradation products detectable by NMR. Chemical species leached from granular PET incubated with consortium 9, consortium 13, or the full consortium were compared to leached products from control samples consisting of UV-treated PET incubated in LCFBM without bacteria.

**FIG 8:**
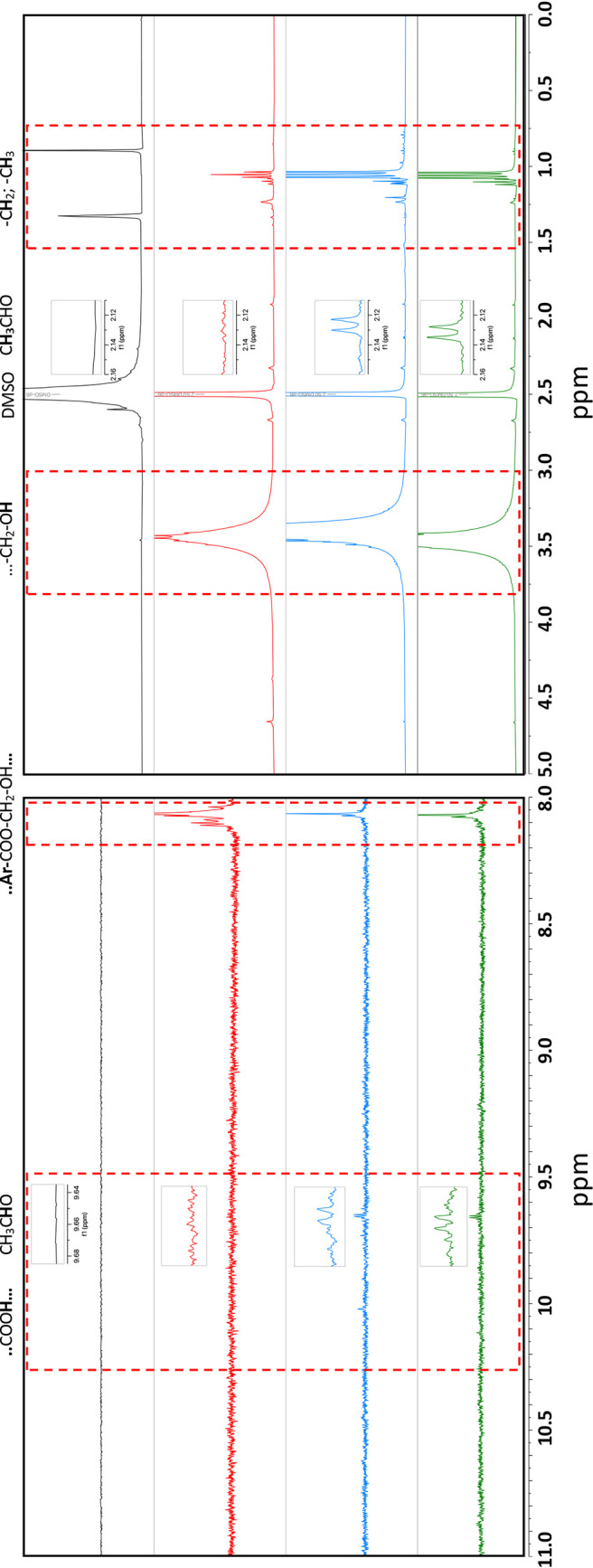
Acetaldehyde, methyl, glycol, and carboxylic acid by-products detected from granular PET treated with consortia. Representative ^1^H NMR spectra from leached granular PET treated with nobacteria (black), full consortium (red), consortium 13 (blue), and consortium 9 (green). Specific regions of interest are highlighted for unique methyl, hydroxyl, aldehyde, and carboxylic acid species. Unique signals are highlighted in red dotted boxes. ^1^H NMR (DMSO-*d*_6_) spectra include 1.04 (t, −CH_3_), 1.88 (m, −CH_2_-CH_3_), 2.12 (d, 3H, acetaldehyde), 2.28 (t, −CH_2_-OH) 3.26 (m, CH_2_-OH), 3.43 (m, CH_2_-OH), 4.28 (m, Ph-CH_2_-CH_2_-OH), 9.66 (q, 1H, acetaldehyde), 10.02 (s, −COOH), 10.87 (s, −COOH).

We observed the generation of unique methyl (1.04 and 1.88 ppm), glycol methylene (3.26 and 3.43 ppm), and hydroxyl (2.28 ppm) end groups in the spectra for the granular PET inoculated with bacteria compared to the uninoculated control (Fig. 8; Table 4). These data were consistent with those observed for the ATR-FTIR analysis (Fig. 7), indicating bacteria-driven ester cleavage of PET plastic. Interestingly, a quadruplet at 9.66 ppm and a distinct doublet at 2.12 ppm integrating at a ratio of 1:3 representing acetaldehyde was uniquely detected in samples treated with bacteria (Table 4). These results suggest the occurrence of the bacterial metabolism of released hydroxyl species from the PET over the detection of random-chain scission events that occur during the generation of PET plastic (29, 30). We observed identical peaks for both consortia 9- and 13-inoculated samples but did not observe the presence of acetaldehyde from the full consortium-treated samples. In addition, we detected nonsignificant levels of the predicted PET hydrolysis products BHET and TPA in the inoculated samples (Table 4), most likely due to the efficient utilization of these degradation products. Further, the full consortium grows more efficiently on PET than consortia 9 and 13 as well as individual isolates (Fig. 3). In addition, while we observed a strong signal for alcohol and glycol methylene groups, a weak signal for carboxylic acid (10.02, 10.87 ppm) was detected from PET samples treated with consortia 9 and 13 only, respectively (Table 4). While weak, potentially due to rapid exchange in a deuterated solvent, the detection of carboxylic acid functionality also provides evidence for the surface hydrolysis of PET by these bacteria.

**TABLE 4:** ^1^H NMR assignments for unique acetaldehyde, methyl, glycol, and carboxylic acid by-products detected from granular PET treated with consortia

Chemical shift^*a*^	Splitting pattern (Hz)	Proton assignment	Consortia^*b*^
1.04	Triplet	−CH_3_ methyl	C9, C13, FC
1.88	Multiplet	CH_2_-CH_3_ methyl	FC
2.13	Doublet	CHOCH_3,_ acetaldehyde^*c*^ (methyl)	C9, C13
2.28	Triplet	−CH_2_-OH	C13, FC
2.50	Pentet	DMSO (solvent)	
3.26	Multiplet	−CH_2_-CH_2_-OH, glycol methylene	C9, C13, FC
3.43	Multiplet	−CH_2_-CH_2_-OH, glycol methylene	C9, C13, FC
4.28	Multiplet	Ph-CH_2_-CH_2_-OH, glycol methylene	FC
9.66	Quadruplet	CHOCH_3,_ acetaldehyde	C9, C13
10.02	Singlet	HCO-COH or -COOH	C13
10.87	Singlet	HCO-COH or -COOH	C9

a H NMR spectra of leached chemical species from granular PET treated with no bacteria was compared to treatment with the full consortium, consortium 13, and consortium 9. Unique chemical shifts upon bacteria treatment are reported.

b For each unique chemical shift, the consortium that generated the signal is reported. C9, consortium 9; C13, consortium, C13; FC, full consortium.

c Validated by standard in DMSO-*d*_6_.

### Genes found to encode various hydrolases and metabolic pathways of PET monomers.

The genomes of the five axenic strains encode numerous α/β-hydrolases, lipases, and esterases (Table 5). The *Pseudomonas* strains 9.2, 10, and 13.2 genomes encode an autotransporter lipase (PlpD), predicted to be associated with the outer membrane (31), and this is consistent with the observed extracellular lipase production (Fig. 2). Also encoded in the *Pseudomonas* genomes is a carboxylesterase, predicted to be secreted (Table S1), that has amino acid sequence similarity to NlhH from the acetyl esterase/lipase superfamily Aes, which hydrolyze short-chain esters, including triacylglycerols and vinyl esters (32). For the cleavage of BHET, the *Bacillus* genomes encode a predicted, secreted (0.7467 Sec/SPII) (33) hydrolase FrsA, where a variant of the enzyme in E. coli cleaves pNP-butyrate (34). The *Pseudomonas* genomes encode a predicted carboxylesterase YpfH, which also cleaves pNP-butyrate (34), but lacked a secretion signal (33). The esterase EstB of *Enterobacter* sp. strain HY1 (35) cleaves BHET, lacks a secretion signal, and also utilizes pNP-butyrate as a substrate. Because pNP-butyrate cleavage has been associated with BHET hydrolysis (34, 35), we concluded that the genomes of both genera, *Bacillus* and *Pseudomonas*, encode BHET cleavage capabilities (Fig. 3 and 6). Through chain scission, UV pretreatment increases the number of soluble surface functionalities on PET, many of which can be hydrolyzed by these enzymes. However, the full consortium was able to grow modestly on amorphous PET film in LCFBM in the absence of UV pretreatment, with culture optical density at 600 nm (OD_600_) values increasing from 0.015 to 0.028 over a 4-week period (*P* = 0.0267), whereas the absorbance did not increase in a culture lacking a carbon source (data not shown). We concluded that a subset of these enzymes (Table 5) or others yet to be identified most likely cleave PET directly.

**TABLE 5:** Strain genomes encode hydrolytic enzymes that may be associated with PET degradation

Enzyme^*a*^ (COG no.[s])	IMG nos.^*b*^	Conserved protein/domain family(ies)	Predicted localization^*c*^	Encoded in strains
Triacylglycerol lipase-like subfamily of the SGNH hydrolases/phospholipase (COG3240, COG4625)	2885786731, 2885796390, 2885806774	EstA, EstP	Outer membrane	9.2, 10, 13.2
Acetyl esterase/lipase (COG0657)	2885789518, 2885793163, 2885803629	NlhH/Aes	Secreted	9.2, 10, 13.2
Bifunctional outer membrane translocase/extracellular lipase (COG1752)	2885788869, 2885795708, 2885806938	PlpD/RssA	Outer membrane	9.2, 10, 13.2
Triacylglycerol esterase/lipase (COG1075)	2885780382, 2886222081	EstA	Secreted	9.1, 13.1
Carboxylesterase (COG0400)	2885787594, 2885795292, 2885805641	YpfH	Cytoplasm	9.2, 10, 13.2
α/β-fold hydrolase (COG1073)	2885779932, 2886223315	FrsA	Secreted	9.1, 13.1

a Lipases, esterases, α/β-fold hydrolases, and carboxylesterases were found to be encoded in all isolates. All isolates shared the conserved protein domain EstA; specifically, *Bacillus* sp. 9.1 and 13.1 encoded a triacylglycerol esterase/lipase, while *Pseudomonas* strains 9.2, 10, and 13.2 encoded a triacylglycerol lipase-like from the SGNH hydrolases subfamily. Only the *Pseudomonas* strain 9.2 encoded an acetyl esterase/lipase from the Aes domain. Only *Pseudomonas* spp. encoded bifunctional outer membrane translocase/extracellular lipase (PlpD) and a predicted carboxylesterase, YpfH, whereas only *Bacillus* spp. encoded an α/β-fold hydrolase, FrsA. Like EstB in *Enterobacter* sp. HY1, FrsA has been shown to display esterase activity on pNP-butyrate, which is correlated with BHET cleavage.

b Genomes/genes annotated via the IMG suite from the Joint Genome Institute (JGI).

c SignalP was used to identify putative signal sequences, indicating secretion.

Enzymes associated with the metabolism of TPA and ethylene glycol were also found to be encoded within the isolates’ genomes (29, 36, 37) (Table 6). Only *Pseudomonas* strains were found to encode transporters and metabolic genes associated with TPA metabolism. While ethylene glycol metabolism can be carried out via several pathways, *Pseudomonas* strains 10 and 13.2 possess all genes encoding ethylene glycol degradation, which proceeds via the acetyl-CoA 3-phosphoglycerate canonical pathway found in Pseudomonas putida KT2400 (37). There was significant overlap in amino acid sequence identity between P. putida KT2400 and strains 10 and 13.2 with respect to the ethylene glycol metabolism-associated enzymes reported in Table 6 (>99%; gap frequency, 0.0%). The *Bacillus* species had diverse metabolic pathways and were able to grow on ethylene glycol as a sole carbon source, but the genes necessary to efficiently utilize TPA are absent. They were unable to grow alone on TPA (data not shown). These data indicated metabolic cooperation by the full consortium in a limited-nutrient environment.

**TABLE 6:** Enzymes associated with ethylene glycol and terephthalic acid metabolism, associated metabolites, and the strains that encode them

Metabolic enzyme(s) (KEGG function no[s].)	IMG nos.	Metabolite(s)^*a*^	Encoded in strains
Alcohol dehydrogenase, *exaA* (K00114)	2885793762, 2885794111, 2885803268, 2885804532	EG	10, 13.2
Glyoxylate carboligase, *gcl* (K01608)	2885790607, 2885796877, 2885807268	EG	9.2, 10, 13.2
Malate synthase, *aceB*, *glcB* (K01638)	2885779017, 2885786797, 2885797786, 2886223009, 2885808276	EG	9.1, 9.2, 10, 13.1, 13.2
Isocitrate lyase, *aceA* (K01637)	2885779016, 2885792116, 2885794657, 2886223008, 2885805160	EG	9.1, 9.2, 10, 13.1, 13.2
4-hydroxythreonine-4-phosphate dehydrogenase pdxA/tphB (K00097, K18076)	2885786748, 2885796374, 2885806758	TPA	9.2, 10, 13.2
Benzoate/toluate 1,2 dioxygenase, *benB xylY* (K05549, K05550)	2885791806, 2885792570, 2885806758	TPA	9.2, 10, 13.2
MFS transporter, AAHS family, 4-hydroxybenzoate transporter, *pcaK* (K08195)	2885787539, 2885789748, 2885792519, 2885795673, 2885803673, 2885806122	TPA	9.2, 10, 13.2
Protocatechuate-3-4-dioxygenase, *pcaG*, *pcaH* (K00448, K00449)	2885789743, 2885794909, 2885805292	TPA, PCA	9.2, 10, 13.2
Catechol-1-2-dioxygenase, *catA* (K03381)	2885791800, 2885792575, 2885797083, 2885804952, 2885807778	TPA, CAT	9.2, 10, 13.2

a Only *Pseudomonas* 10 and 13.2 encode all genes associated with EG metabolism. *Pseudomonas* strains 9.2, 10, and 13.2 encode enzymes responsible for TPA metabolism/downstream metabolites protocatechuate (PCA) and catechol (CAT), while neither *Bacillus* strains encode this capability. EG, ethylene glycol; TPA, terephthalic acid.

## DISCUSSION

Due to their highly stable polymeric structure, postconsumer plastics do not degrade easily in the environment, and strategies must be implemented to assist and enhance their degradation to prevent plastic accumulation. It is logical that *Pseudomonas* species, which are abundant in soils, are a viable option to biodegrade plastic due to their ability to degrade a wide range of compounds, both aliphatic and aromatic (17, 38). Additionally, since several species of *Pseudomonas* and *Bacillus* generate and metabolize their own biopolymers, such as polyhydroxyalkanoic acids (PHAs), this suggests they possess the ability to utilize polymers as carbon sources (39, 40). To date, researchers have identified a limited number of *Pseudomonas* species that degrade PET (41, 42). Although a cutinase from P. mendocina was shown to have a high affinity for low-crystalline PET, most *Pseudomonas* species have shown more aliphatic polyesterase activity as opposed to aromatic polyesterase activity (43).

In this report, evidence for PET degradation by bacterial consortia containing both *Pseudomonas* and *Bacillus* species includes growth on PET as a sole carbon source (Fig. 3), surface modification determined by SEM and ATR-FTIR (Fig. 5 and 7), and hydrolysis using ^1^H NMR spectroscopy (Fig. 8). The holes observed by SEM in Fig. 5E and F are reminiscent of those observed for low-crystallinity PET incubated with the purified cutinase HiC from the fungus Humilica insolens (15). Within the *Pseudomonas* genomes, we identified two outer membrane-associated autotransporter lipases, EstP and PlpD (Table 5), that could account for amorphous PET surface degradation, and the tight attachment of the *Pseudomonas*, even after washing with detergent, that was observed in Fig. 5. These data are consistent with our previous published observation that after 8-weeks incubation, the full consortium reduced the weight of granular PET pellets by 3% (26).

Several researchers have suggested using multiple enzyme systems, whole microbes, or consortia of bacteria for the biodegradation of PET plastic (10, 44). With two enzymes (45) and/or two or more bacterial species, even difficult-to-degrade compounds can be mineralized. Perhaps predictably, we were unable to detect significant quantities of the degradation products BHET or TPA by ^1^H NMR, most likely because BHET is efficiently degraded by enzymes secreted by the *Bacillus* species and by the consortia bacteria (Fig. 8B), and TPA is readily utilized for growth by the *Pseudomonas* species (Table 6). Further, we detected hydroxyl peaks with significant quantities of acetaldehyde, most likely derived from the PET monomer ethylene glycol, and there are several possible explanations for the presence of acetaldehyde. After bacteria-driven hydrolysis generating ethylene glycol, conversion of ethylene glycol to acetaldehyde can occur under acidic conditions without enzyme catalysis (46). Second, the acetaldehyde was generated by bacterial alcohol dehydrogenases converting ethylene glycol to acetaldehyde. All five of our consortium member genomes encode alcohol dehydrogenases (Table S2 in the supplemental material), and the presence of small alcohol groups to convert to acetaldehyde is indicative of plastic degradation (29). Lastly, during the processing of PET, thermal degradation causes random scission of the in-chain ester linkage resulting in the formation of a vinyl ester and carboxyl end groups (47). The hydrolysis of these groups can lead to the formation of acetaldehyde. The presence of acetaldehyde in only the bacteria-treated, but not in leached PET samples (Fig. 8), suggested that this did not occur, rather that the acetaldehyde resulted from bacterial enzyme catalysis.

Evidence supporting synergistic degradation included significantly increased lipase enzymatic activity when grown in consortia as opposed to single strains (Table 1), along with increased growth rates on PET and BHET when grown in a full consortium (Fig. 3) and complete conversion of BHET to TPA by enzymes secreted by the full consortium (Fig. 6B). From initial analysis of the genome sequences (26) and predicted differential metabolic pathways (29, 36), the ability of the *Pseudomonas* species to degrade the PET cleavage product TPA (Table 6) was present in all three *Pseudomonas* strains. While the *Bacillus* species do not encode TPA degradation genes, they do encode ethylene glycol metabolism (Table 6). This, coupled with the secreted enzymes responsible for significantly converting BHET to TPA (Fig. 6), potentially via the encoded hydrolase FrsA, supports the observed synergy between the two species. The presence of the cytoplasmic *Pseudomonas* YpfH, lacking a secretion signal, which is functionally similar to the recently described *Enterobacter* sp. HY1 EstB able to cleave BHET (35), may explain why strains 9.2, 10, and 13.2 grew on BHET (Fig. 3), while secreted enzymes from these strains did not significantly degrade this compound (Fig. 6). *Bacillus* strains 9.1 and 13.1, were able to cleave the polyester polycaprolactone, Tween 20, and Tween 80, while *Pseudomonas* species possessed medium- and long-chain fatty acid degradation capabilities (Table 2). These data illustrate that enhanced growth of the full consortium on PET as a sole carbon source (Fig. 3) is most likely due to mixed enzymatic activities. The synergistic capabilities of a mixed culture of *Pseudomonas* and *Bacillus* capable of degrading PET monomers were observed in previous research by Kimura and Ito of Pioneering Research Laboratories, Toray Industries Inc. (19). Kimura and Ito discovered a species of *Pseudomonas* capable of degrading TPA, and a *Bacillus* that could not that, when mixed, were able to clear 99% of TPA from polyester manufacturing wastewater and were also unaffected by ethylene glycol accumulation. Our results extend Kimura’s and Ito’s findings in their mixed culture of *Pseudomonas* and *Bacillus* isolates that degrade the PET by-product TPA.

Some mechanistic studies have described how certain bacteria and fungi are able to utilize PET as a sole carbon source. Reported products of PET biodegradation are BHET and MHET, and PETase and MHETase enzymes necessary for this process have been described for the bacterium Ideonella sakaiensis (2). In our study, it was difficult to identify which secreted enzymes from the five strains were responsible for the chemical modification of PET, in part because there are a considerable number of lipases and related enzymes encoded within the genomes of these bacteria (Table 5). Experimentally, we attempted to precipitate and to identify secreted enzymes when the bacteria, particularly the full consortium, were grown in the presence of PET. Possibly due to biofilm formation and/or that secreted enzymes were bound directly to the hydrophobic surface of the plastic surface, which has been observed previously (29, 43), we were unable to identify secreted enzymes involved directly in PET degradation.

Viable PET plastic biodegradation strategies will most likely require the concerted effort of consortia of bacteria. Given the many types of plastics to degrade (14), and the possibility of forming complex communities that can degrade plastic faster than individual species alone, well-formulated consortia for this purpose are a desirable strategy (10). It was previously shown that a naturally selected low-density and high-density polyethylene-degrading consortium of bacteria can degrade these plastics faster than individual strains of the consortium (48). Further, consortium-based approaches to bioaugmentation-based pollution cleanup have been successfully utilized before on a monumental scale, such as in the aftermath of the Deepwater Horizon oil spill (49). In the future, it may be possible to utilize a range of consortia bacteria to degrade different polyesters and to purify key metabolic by-products that can be exploited as valuable chemicals. This approach could lead to biorecycling and drive a more circular plastics economy, reducing plastic pollution worldwide.

## MATERIALS AND METHODS

### Soil collection.

There are 11 superfund sites that exist in the greater Houston, Texas, area (50). Using the logic that bacteria in polluted environments are more likely to adapt to harnessing pollutants to survive, soil samples (∼500 g) were collected from the Gulf Coast of southeast Texas, within the greater Houston area, and near the shoreline at East Beach in Galveston. The samples were collected roughly 6 in. beneath the topsoil layer and refrigerated prior to transport to Portland, Oregon.

### Isolating bacteria.

Each soil sample (2 g) was resuspended in 9 ml phosphate-buffered saline (PBS) prepared as follows: 8 g NaCl, 0.2 g KCl, 1.44 g Na_2_HPO_4_, and 0.24 g KH_2_PO_4_ per 1 liter deionized water (diH_2_O), adjusted to pH 7.4 and autoclaved for 20 min (15 lb/in^2^, 121°C). The soil and PBS suspensions were placed on a rotary shaker (250 rpm) for 24 h. The sediment was allowed to settle, and 100 μl of this suspension was then spread on lysogeny broth (LB) agar prepared as follows: 10 g tryptone, 5 g yeast, 5 g NaCl, and 18 g agar per 1 liter diH_2_O, adjusted to pH 7.0 and autoclaved (15 lb/in^2^, 121°C). Multiple plates were made from each soil sample, inverted, and incubated at 26°C for 24 h.

### Lipase screening.

Rhodamine B agar plates (9.0 g nutrient broth powder, 2.5 g yeast extract, and 10 g agar per 1 liter H_2_O) were prepared to test isolated bacterial colonies for lipase activity. For the lipid emulsion media, 250 μl of Tween 80 and 30 ml olive oil were added to 50 ml diH_2_O and emulsified in a blender. The final lipoidal emulsion was adjusted to pH 7. The base medium and lipoidal emulsion were autoclaved separately. Following autoclaving, rhodamine B was added to a concentration of 0.024% (wt/vol) to the sterile lipoidal emulsion. Lipoidal emulsion (50 ml) was then added to the base nutrient media to a final volume of 1 liter and mixed thoroughly, with the final concentration of rhodamine B at 0.0012% (wt/vol) and then poured (51).

Colonies grown on LB agar were screened for bacterial lipolytic activity via a colony lift assay from the LB plate to rhodamine B agar. The rhodamine B plates were inverted and incubated for 24 hours at 26°C. Colonies producing lipase on rhodamine B agar were identified as those that produced fluorescent halos when exposed to a UV trans-illuminator at 365 nm and were restreaked onto individual LB plates for isolation and purification (see Fig. 1). E. coli strain MC4100 was used as a negative control. This assay was repeated to ensure isolated strains remained lipase positive. In sum, 192 colonies were screened for lipase activity on rhodamine B plates. One hundred seventy-eight were negative, with 14 colonies initially being positive. Three colonies remained lipase positive on rhodamine B agar.

### Strain deposition.

The following bacterial strains were deposited with the USDA NRRL Culture Collection: *Pseudomonas* sp. SWI36, NRRL B-67630, strain 10; Bacillus albus strain PFYN0, NRRL B-67631, strain 13.1; Bacillus thuringiensis strain C15, NRRL B-67632, strain 9.1; *Pseudomonas* sp. strain B10, NRRL B-67633, strain 9.2; and *Pseudomonas* sp. SWI36, NRRL B-67634, strain 13.2.

### General hydrolytic and polyesterase activity screening.

General hydrolytic and polyesterase activities were examined via tributyrin, coconut oil, and polycaprolactone diol (*M*_n_, 530) (PLD) substrate agar plates according to Molitor et al. (52) to examine esterase and lipase activity in addition to the olive oil assay described above. Tributyrin was used to screen for all general hydrolytic activities, including polyesterases, esterases, lipases, and acyltransferases. Plates were inoculated and grown at 20°C for 5 days, while coconut oil was used as a medium-chain fatty acid to screen for more specific lipase and esterase activity, and they were grown at 26°C for 2 days. Polyesterase activity was confirmed by grainy hydrolytic by-products surrounding colonies using the aliphatic polyester polycaprolactone diol (*M*_n_, 530) as a substrate on LCFBM agar plates after growth of inoculated strains at 20°C for 5 days (52). Strains were also inoculated on CaCl_2_-Tween 20 or CaCl_2_-Tween 80 agar plates and grown for 4 days (CaCl_2_) at 26°C. CaCl_2_ plates were screened in ambient light, and precipitation around the point of inoculation is indicative of esterase activity on the CaCl_2_ agar.

### Bacterial growth curves.

For monitoring bacterial growth on PET and BHET, strains and consortia bacteria were inoculated from preconditioned culture with either 0.1 g of PET or BHET in a 1.5-cm glass test tube containing 10 ml of the following medium (in 1 liter): 0.05% (wt/vol) yeast extract, 0.2% (wt/vol) (NH_4_)_2_SO_4_, and 1% (vol/vol) trace elements (0.1% [wt/vol] FeSO_4_·7H_2_O, 0.1% [wt/vol] MgSO_4_·7H_2_O, 0.01% [wt/vol], CuSO_4_·5H_2_O, 0.01% [wt/vol] MnSO_4_·5H_2_O, and 0.01% [wt/vol] ZnSO_4_·7H_2_O) in 10 mM sodium phosphate buffer (pH 7.4). Then, for growth experiments where PET was the sole source of carbon, liquid carbon-free base medium (LCFBM) was prepared in the absence of yeast extract as follows: 0.2% (wt/vol) (NH_4_)_2_SO_4_ and 1% (vol/vol) trace elements (0.1% [wt/vol] FeSO_4_ 7H_2_O, 0.1% [wt/vol] MgSO_4_·7H_2_O, 0.01% [wt/vol] CuSO_4_·5H_2_O, 0.01% [wt/vol] MnSO_4_·5H_2_O, and 0.01% [wt/vol] ZnSO_4_·7H_2_O) in 10 mM sodium phosphate buffer (pH 7.4) per 1 liter of diH_2_O (2). LCFBM was supplemented with 1% (wt/vol) of PET granules (Sigma-Aldrich, St. Louis, MO) that were pretreated overnight with UV irradiation (250 nm) or with 10 mM BHET (Sigma-Aldrich, St. Louis, MO). Amorphous PET film was purchased from Goodfellow Corporation, Coraopolis, PA.

Bacteria from preconditioning cultures described above were pelleted, washed with PBS, pH 7.4, and resuspended to equal OD_600_ values before addition in triplicate to plastic-supplemented LCFBM (starting OD_600_ of 0.02). Samples with BHET were incubated in an environmental shaker (30°C, 125 rpm), and samples with PET were incubated statically to facilitate biofilm formation at 30°C. Subsequent OD_600_ values were monitored daily. Negative controls with no plastic supplementation were monitored for growth, and cultures with no inoculation were used as blanks.

Growthcurver, an R package that calculates metrics for microbial growth curves, was used to generate doubling times and carrying capacities for each culture condition (53). Growthcurver fits a logistic equation (N_t_ = *K*/{1 + [(*K* − *N*_0_)/*N*_0_]e^−rt^} (where N_0_ is the initial population size, K is the carrying capacity, r is the intrinsic growth rate, t is time, and N_t_ is the number of cells at a given time) to the experimentally obtained curve data. Smaller values of the residual sum of squares, σ, from the nonlinear regression models indicated a better fit of the logistic curve to the data. *P* values of <0.05 were considered significant.

CFU were used as a metric for attachment efficiency of each isolate. Two different PET substrates, (0.1 g) postconsumer water bottles (Desani) or (0.1 g) PET granules without additives (Sigma-Aldrich, St. Louis, MO) in LCFBM were inoculated with approximately 10^3^ CFU of each strain and incubated at 30°C for 6 weeks. After 6 weeks incubation, CFU values for each culture condition on PET were collected by washing pellets with sterile MilliQ water, lightly vortexing postincubated pellets in 0.2% saponin in liquid carbon-free media, and plating serial dilutions on LB in experimental triplicates. To ensure that the saponin did not lyse the cells open, 0.2% saponin was added to cultures of each isolate in liquid carbon-free media with 0.1% glucose. A standard plate count comparison of these cultures with and without saponin added showed that the isolates were not lysed in the presence of 0.2% saponin. Negative controls of substrate in media with no inoculation were plated to determine if there was any contamination.

### Enzyme harvest and NMR analysis of BHET degradation.

For harvesting secreted enzymes to illustrate BHET degradation (see Fig. 6), LCFBM with 10 mM BHET was inoculated with strains 9.1; 9.2; consortia 9, 10, 13.1, or 13.2; consortium 13; or the full consortium and incubated for 3 weeks (200 ml total for each culture). Following the incubation period, cultures were at an average OD_600_ of 0.3 and centrifuged at 13,000 rpm for 20 min at 4°C to remove bacteria. Culture supernatants were sterile filtered, concentrated, and normalized by Bradford assay to the lowest concentration (1 mg/ml). The full volume was added to fresh BHET substrate (10 mM) and placed at 30°C for 4 weeks to determine BHET degradation. Resulting precipitates from the enzymatic degradation were extracted by centrifugation, dried to remove water, resuspended in DMSO-*d*_6_ (Sigma-Aldrich, St. Louis, MO), and analyzed by ^1^H NMR with an Avance Generation I spectrometer operating at 400 mHz. Standards of BHET and TPA in DMSO-*d*_6_ were used to identify resulting peaks. Reference BHET peaks at 3.86 ppm (4H) were used to find the ratio of TPA (8.03 ppm) to BHET (8.07 ppm) by integration for each experiment.

### Postconsumer PET surface characterization using ATR-FTIR.

Surface modification of postconsumer PET water bottle fragments, with and without UV irradiation, was performed as follows. Culture tubes were filled with 8 ml LCFBM (0.7 g of KH_2_PO_4_, 0.7 g of K_2_HPO_4_, 0.7 g of MgSO_4_·7H_2_O, 1.0 g of NH_4_NO_3_, 0.005 g of NaCl, 0.002 g of FeSO_4_·7H_2_O, 0.002 g of ZnSO_4_·7H_2_O, and 0.001 g of MnSO_4_·H_2_O per 1 liter of diH_2_O) (54) and inoculated with 50 μl overnight culture, normalized to an OD_600_ of 1.0. Overnight cultures in LB with consortium 9 and 13 and strain 10 or E. coli MC4100 as a negative control were centrifuged and washed with PBS prior to inoculation. The LCFBM cultures contained 2.0% (wt/vol) 70% ethyl alcohol (EtOH)-sterilized postconsumer PET strips (25 by 5 mm strips) as the sole carbon source. Half of the PET samples were pretreated with UV radiation (365 nm for 30 min) to enhance hydrophilicity and enzyme attachment by increasing carbonyl or hydroxyl functionality of the substrate (5, 6). Tubes were set up in triplicate and incubated without shaking at 26°C for 6 weeks. Samples were replenished with sterile LCFBM each month to counter evaporation. Plastic PET strips were then submerged in 30 ml 2% SDS and placed on the rotary shaker for 2 h (225 rpm, 37°C) to remove adherent bacteria, immersed in fresh diH_2_O water, and air-dried. For ATR-FTIR analysis, a Thermo Scientific iS5 infrared spectrometer and iD7 diamond-ATR attachment were used to acquire spectra from 4,000 to 450 cm^−1^ (4 cm^−1^ resolution) with OMNIC software. Data were transformed using an N-B strong apodization and Mertz phase correction. Three areas were analyzed for each sample to obtain spectra that represent the average condition of the plastic surface. All infrared spectra were normalized by peak intensity to common C-H bending modes used for PET, 1,409 cm^−1^.

Peak intensity ratios for ATR-FTIR were calculated using the average intensities from nine spectra (3 measurements × 3 samples for each condition), and standard deviations were used to propagate errors prior to calculation of confidence intervals (*n* = 9; α = 0.05, where α is the cutoff level for significance). Studies performed in biological triplicate were compared by unpaired two-tailed Student's *t* test or 1-way analysis of variance (ANOVA) with replication and Tukey’s HSD. Values of *P < *0.05 were considered significantly different.

### Scanning electron microscopy.

UV-pretreated, amorphous PET (Goodfellow Corporation) was inspected for degradation after culturing with the full consortium incubated at 30°C in LCFBM for 40 days. The PET plastic samples were removed from media and rinsed twice in a 0.1% saponin solution followed by sterile molecular biology-grade water via vortexing. After rinsing, a 70% ethanol rinse was used to sterilize all surfaces of plastic. Samples were dried for 24 h before imaging. Half of the samples were immediately imaged after drying, while half were treated with a 100-μg/ml proteinase K treatment to remove tightly adherent bacteria and then sterilized again in 70% ethanol prior to imaging. Samples were submerged in 2% osmium tetraoxide in an ice bath for 3 h. The samples were then dehydrated in graded EtOH (50, 75, and 100%) baths for 15 min each before undergoing critical point drying with CO_2_. Dried samples were sputter coated with gold using a Leica ACE600 coater prior to imaging with an FEI Helios Nanolab 660 DualBeam focused ion beam (FIB)-SEM and thermoluminescent dosimeter (TLD) detector operating at an accelerating voltage of 2 kV with 1 μs dwell time. SEM was performed at the Multi-Scale Microscopy Core (MMC) with technical support from the Oregon Health and Science University (OHSU)/FEI Living Lab and the OHSU Center for Spatial Systems Biomedicine (OCSSB).

### NMR analysis of PET degradation.

Consortia bacteria were first diluted to an OD_600_ of 1 in sterile PBS, and 50 μl was added per 5 ml culture tubes with UV pretreated 2% (wt/vol) amorphous PET (Goodfellow Corporation) in a culture flask in LCFBM. Samples were incubated statically at 26°C. After 4 weeks of incubation, tubes were vortexed briefly, and then the medium was sterilized using a filter flask, collected for media NMR analysis, and kept in sterile culture tubes. PET was rinsed in a dilute saponin solution (0.01% wt/vol) to remove bacteria from the surface of PET and then sterilized with 70% EtOH. After complete EtOH evaporation, PET was then placed in 600 μl DMSO-*d*_6_ with 0.03% trimethylsilyl (TMS) (Sigma-Aldrich, St. Louis, MO) and placed in a sonicator bath for 1 h to leach biodegradation by-product from the surface of the plastic. Solutions were placed in NMR tubes. Standards of TPA, BHET, and PET were prepared in DMSO-*d*_6_ with 0.03% TMS. An 800-MHz NMR spectrometer at the NMR Facility at Oregon State University was utilized for one-dimensional NMR analysis using a zg30 pulse sequence (single, 30-degree proton pulse) to enhance signal to noise. Spectra were visualized and analyzed using MestReNova (Mnova) software (55).

### Genetic and metabolic pathway analysis.

Draft genomes of all five strains (BioProject accession no. PRJNA517285) were annotated using the JGI-IMG/MER database (56) and analyzed for hydrolytic enzymes that may induce PET degradation, including lipases, esterases, hydrolases, and cutinases. All genomes were stored in Genomes Online Database (GOLD) (57) under study IDs Gs0149331 (*Pseudomonas* sp. 9.2, 10, and 13.2) and Gs0149339 (*Bacillus* sp. 9.1 and 13.1). Putative enzymes were evaluated using NCBI’s Conserved Domain Database (58). Metabolic pathways for PET monomers were analyzed using KEGG (39). Protein signal peptides were predicted with SignalP (33) using default parameters.

10.1128/mSphere.01151-20.1 TABLE S1 Predicted secretion signals of putative hydrolytic enzymes determined using SignalP 5.0. Download Table S1, PDF file, 0.1 MB. Copyright © 2020 Roberts et al. 2020 Roberts et al. This content is distributed under the terms of the Creative Commons Attribution 4.0 International license.

10.1128/mSphere.01151-20.2 TABLE S2 Enzymes encoded in the genomes implicated in ethylene glycol and acetaldehyde metabolism. Download Table S2, PDF file, 0.1 MB. Copyright © 2020 Roberts et al. 2020 Roberts et al. This content is distributed under the terms of the Creative Commons Attribution 4.0 International license.

10.1128/mSphere.01151-20.3 FIG S1 BHET is hydrolyzed by secreted enzymes generating ethylene glycol and terephthalic acid. Consortium 9 and the full consortium were grown with BHET substrate as the only carbon source. Native secreted proteins were then collected from filtered culture supernatants. The full collection of secreted proteins were incubated with fresh BHET substrate. Resulting insoluble degradation products were analyzed by ^1^H NMR in DMSO-*d*_6_ for comparison to an untreated control. A glycol methylene triplet of BHET (3.73 ppm) was used as the reference peak for 4 hydrogens. BHET uninoculated (A), BHET treated with secreted proteins from consortium 9 (B), and the full consortium (C). Note peak at 13.28 ppm (1H, s, TPA carboxylic acid) absence in the uninoculated control (A), with increasing intensity when inoculated with consortium 9 (B) and then the full consortium (C). Download FIG S1, PDF file, 1.3 MB. Copyright © 2020 Roberts et al. 2020 Roberts et al. This content is distributed under the terms of the Creative Commons Attribution 4.0 International license.
