# Supplementary material for: Environmental Consortium Containing Pseudomonas and Bacillus Species Synergistically Degrades Polyethylene Terephthalate Plastic
Source: mSphere. 2020 Dec 23;5(6):e01151-20. doi: 10.1128/mSphere.01151-20 (PMC7763552; doi:10.1128/mSphere.01151-20)
Supplement: TABLE S1 [file mSphere.01151-20-st001.pdf]

Table S1. Predicted secretion signal likelihood of putative hydrolytic enzymes

Enzyme / Genus / COG ID	Signal Peptide Likelihood[1]	Predicted secretion signal
NlhH / *Pseudomonas* / COG0657	0.9983	MNIKKTLAASLLALSIGNAFA
EstA / *Bacillus* / COG1075	0.9958	MRLMRRCVALLIVFFIMAPTISTNVQA
PlpD / *Pseudomonas* / COG1752	0.9909, 0.9835	MRRLLFCFLLVLTSLTAIA
FrsA / *Bacillus* / COG1073	0.7467*	LKRFKFYMISGIVLVLLI
EstP / *Pseudomonas* / COG3240	0.7259	MRKAPLLRFTLASLALACSQALA
EstA / *Pseudomonas* / COG4625	0.6909	MIKQTLFVPLAGCLLAMACAQANA

[1]The SignalP 5.0 server utilizes a deep convolutional and recurrent neural network architecture to predict the presence and location of cleavage of Sec/SPI, Sec/SPII*, and Tat/SPI signal peptides.
