# Supplementary material for: Environmental Consortium Containing Pseudomonas and Bacillus Species Synergistically Degrades Polyethylene Terephthalate Plastic
Source: mSphere. 2020 Dec 23;5(6):e01151-20. doi: 10.1128/mSphere.01151-20 (PMC7763552; doi:10.1128/mSphere.01151-20)
Supplement: TABLE S2 [file mSphere.01151-20-st002.pdf]

Table S2. Encoded enzymes implicated in ethylene glycol and acetaldehyde metabolism

**Strain**[1]	**Encoded alcohol dehydrogenases (IMG ID)**	**Encoded aldehyde dehydrogenases (IMG ID)**
9.1	Alcohol dehydrogenase (2885778482, 2885778935)	Aldehyde-alcohol dehydrogenase (2885777480)
9.2	Alcohol dehydrogenase (2885787358)	NAD-dependent aldehyde dehydrogenase (2885787734)
10	Alcohol dehydrogenase (cytochrome c) (2885793762)	Acyl-CoA reductase-like NAD-dependent aldehyde dehydrogenase (2885792959)
13.1	Alcohol dehydrogenase (2886220319, 2886222383)	Aldehyde-alcohol dehydrogenase (2886219295)
13.2	Alcohol dehydrogenase (cytochrome c) (2885803268)	NADP-dependent aldehyde dehydrogenase (2885803149, 2885803261)

[1]Strain 9.1 *B. thuringiensis* strain C15; strain 9.2 *Pseudomonas* sp. B10; strains 10 and 13.2 *Pseudomonas* sp. SWI36; and strain 13.1 *Bacillus albus*.
