# Supplementary material for: Environmental Consortium Containing Pseudomonas and Bacillus Species Synergistically Degrades Polyethylene Terephthalate Plastic
Source: mSphere. 2020 Dec 23;5(6):e01151-20. doi: 10.1128/mSphere.01151-20 (PMC7763552; doi:10.1128/mSphere.01151-20)
Supplement: FIG S1 [file mSphere.01151-20-sf001.pdf]

# A. Uninoculated

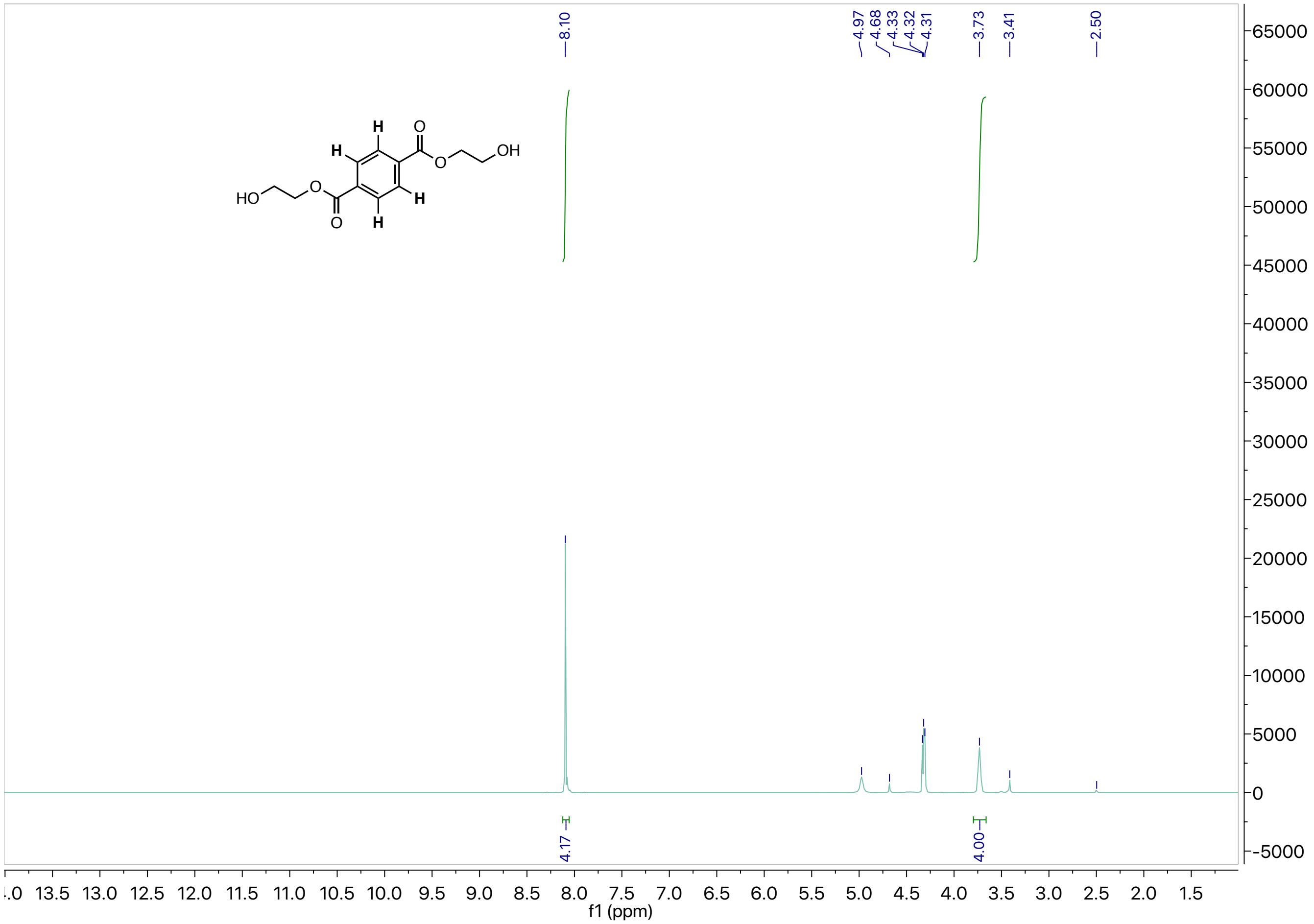


$^{1}$H NMR (d6-DMSO): 2.50 (p), 3.40 (s), 3.72 (4H, s, J = 4.0, 4.0 Hz, BHET methylene), 4.31 (4H, s, J = 4.0, 4.0 Hz, BHET methylene), 4.67 (s), 8.08 (s, BHET aromatic).

## B. Consortium 9

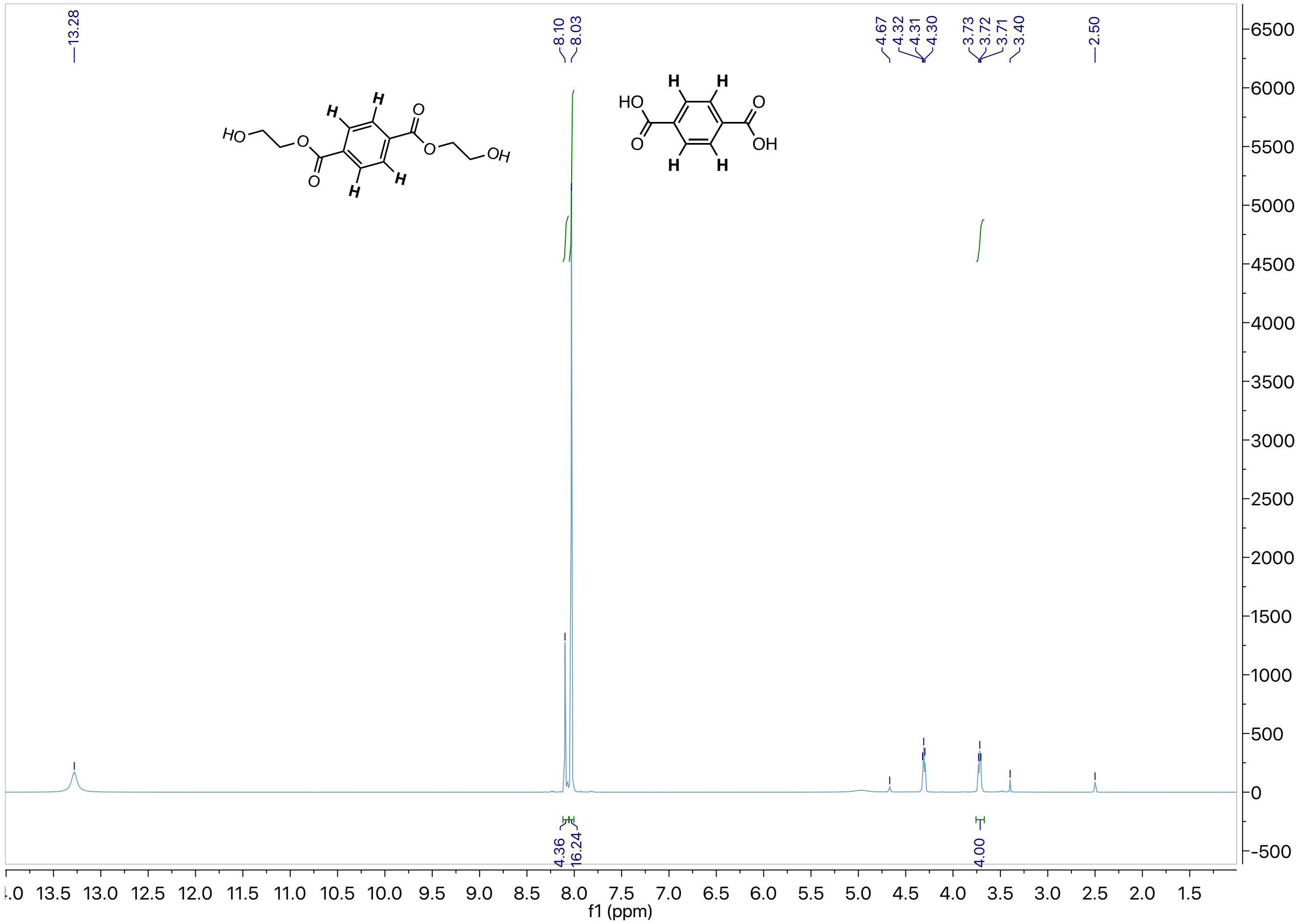


$^1$H NMR (d6-DMSO): 2.50 (6H, p), 3.40 (s), 3.72 (4H, s, J = 4.0, 4.0 Hz, BHET methylene), 4.31 (4H, s, J = 4.0, 4.0 Hz, BHET methylene), 4.67 (s), 8.03 (4H, s, TPA aromatic), 8.10 (4H, s, BHET aromatic), 13.28 (1H, s, TPA carboxylic acid).

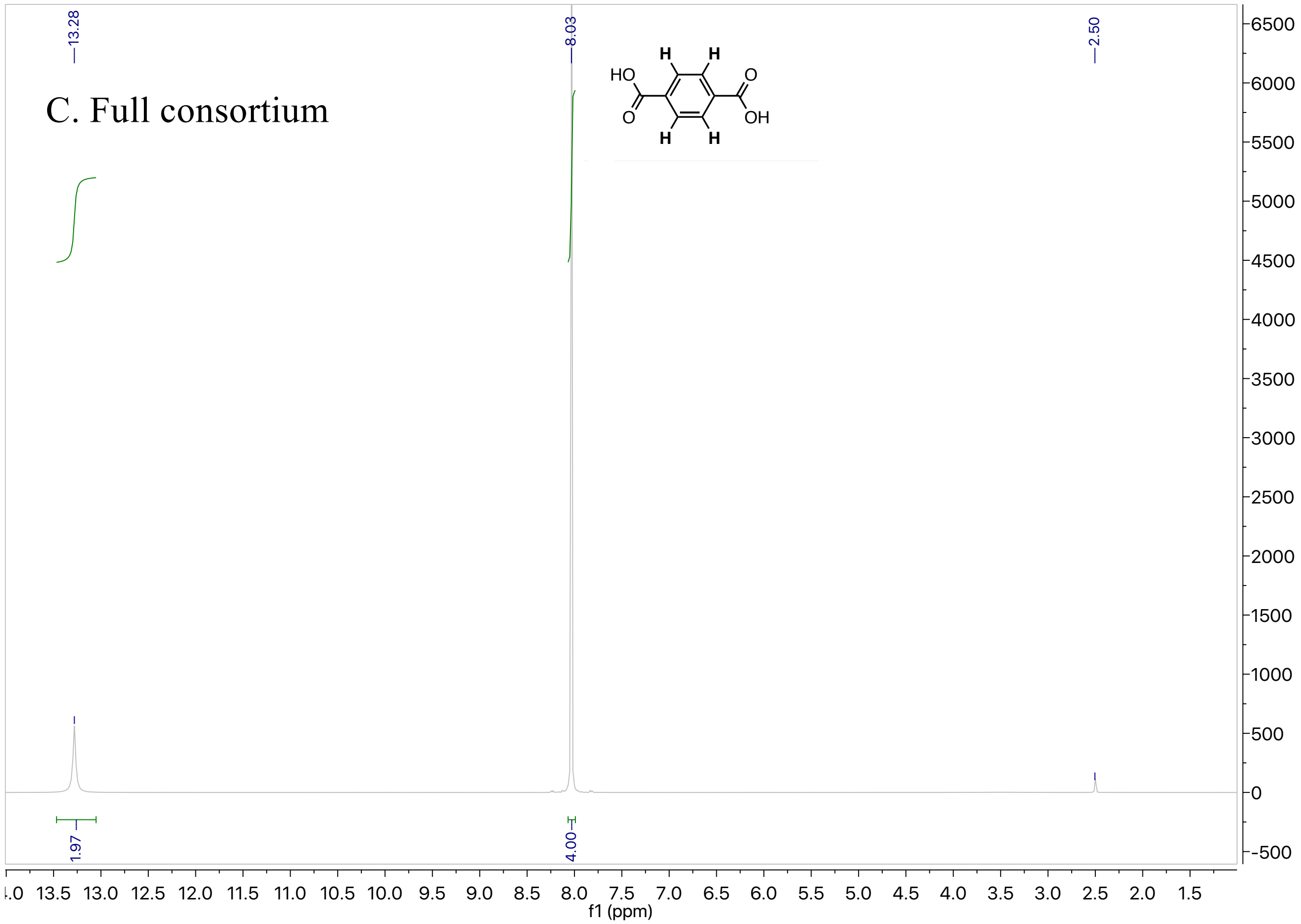


$^1$H NMR (DMSO-$d_6$): 2.50 (6H, p, DMSO), 3.40 (s), 3.72 (4H, s, J = 4.0, 4.0 Hz, BHET methylene), 4.31 (4H, s, J = 4.0, 4.0 Hz, BHET methylene), 4.67 (s), 8.03 (4H, s, TPA aromatic), 8.10 (4H, s, BHET aromatic), 13.28 (1H, s, TPA carboxylic acid).
